# Retinal Perfusion and Injury in Sepsis and after Major Surgery

**DOI:** 10.1016/j.xops.2025.100890

**Published:** 2025-07-22

**Authors:** Ella Courtie, Gagana Mallawaarachchi, Aditya U. Kale, Ahmed Gilani, Nicholas Capewell, Donna Holding, Benjamin T.K. Hui, Xiaoxuan Liu, Elinor Laws, Ann Logan, Tony Whitehouse, Alastair K. Denniston, Tonny Veenith, Richard J. Blanch

**Affiliations:** 1 Neuroscience and Ophthalmology Research Group, University of Birmingham, Birmingham, UK; 2 University Hospitals Birmingham NHS Foundation Trust, Department of Ophthalmology, Queen Elizabeth Hospital Birmingham, West Midlands, UK; 3 Surgical Reconstruction and Microbiology Research Centre, University Hospitals Birmingham NHS Foundation Trust, Birmingham, UK; 4 Institute of Inflammation and Ageing, University of Birmingham, Birmingham, UK; 5 NIHR Birmingham Biomedical Research Centre, University Hospitals Birmingham NHSFT, Birmingham UK; 6 Ophthalmology Department, Royal Victoria Hospital, Belfast, UK; 7 Critical Care Unit, University Hospitals Birmingham NHS Foundation Trust, Queen Elizabeth Hospital Birmingham, Birmingham, UK; 8 Axolotl Consulting Ltd., Worcestershire, Droitwich, UK; 9 Division of Biomedical Sciences, Warwick Medical School, University of Warwick, UK; 10 NIHR Biomedical Research Centre for Ophthalmology, Moorfields Eye Hospital NHS Foundation Trust and UCL Institute of Ophthalmology, London, UK; 11 Department of Trauma Sciences, University of Birmingham, Birmingham, UK; 12 Academic Department of Military Surgery and Trauma, Royal Centre for Defence Medicine, Birmingham, UK

**Keywords:** OCT, OCTA, Retinal perfusion, Sepsis, GCL

## Abstract

**Objective:**

Assess retinal perfusion in sepsis, compared with uncomplicated postoperative care and healthy controls, and assess the effects of reduced perfusion on retinal structure and visual function.

**Design:**

We conducted a prospective observational cohort study between March 2018 and December 2022, with follow-up measures collected 3 to 6 months after discharge.

**Subjects:**

Twenty-four patients with sepsis were assessed in the intensive care unit (ICU) and 3 to 6 months later, 45 ICU control patients assessed during elective ICU admission after upper gastrointestinal cancer surgery, preoperatively, and 3 to 6 months later, and 15 healthy controls.

**Testing:**

Assessments included retinal layer thickness using OCT, retinal perfusion using OCT angiography, and visual function using Humphrey visual field analysis. Organ dysfunction was assessed by Sequential Organ Failure Assessment (SOFA) scoring.

**Main Outcome Measures:**

Superficial vascular plexus (SVP) retinal perfusion, OCT retinal ganglion cell layer (GCL) thickness, and mean deviation (MD) on Humphrey visual field testing were evaluated.

**Results:**

Superficial vascular plexus retinal perfusion was 37.4% lower in patients with sepsis compared with ICU control patients (*P* < 0.001) and 59.7% lower than in healthy controls, which returned to normal by final follow-up. Retinal perfusion correlated with the SOFA score (Pearson *r* = -0.57, *P* < 0.001) and weakly correlated with C-reactive protein (*r* = -0.337, *P* = 0.01) and mean arterial pressure (*r* = 0.354, *P* = 0.006). In patients with sepsis and ICU controls, retinal perfusion in the ICU predicted subsequent GCL thickening, with every 1-unit decrease in SVP sum predicting a 1.88 μm increase in GCL thickness at follow-up (*P* = 0.003), and worsening visual field MD, with every 1-unit decrease in SVP sum predicting a 0.078 decibel lower MD (*P* = 0.023).

**Conclusions:**

Retinal perfusion was impaired in patients with sepsis compared with both healthy controls and patients after major surgery. It was moderately associated with other measures of organ dysfunction assessed by SOFA. Reduced retinal perfusion in both patients with sepsis and patients after major surgery is strongly associated with subsequent GCL thickening and less strongly associated with decreased visual field MD, suggesting reduced retinal perfusion is associated with retinal damage, with consequent visual dysfunction.

**Financial Disclosure(s):**

Proprietary or commercial disclosure may be found in the Footnotes and Disclosures at the end of this article.

Sepsis is a dysregulated inflammatory response to infection that leads to potentially fatal organ dysfunction.^1^ Globally, an estimated 11 million people die from sepsis annually.^2^ Patients with sepsis may suffer hemodynamic instability and reduced microcirculatory perfusion,^3^ compromising oxygen delivery to vital organs, such as the brain, eyes, heart, and kidneys.^4^

The microcirculation normally ensures a steady blood supply to tissues as systemic macrocirculatory parameters vary, through local autoregulation.^5^ Organs with low ischemic tolerance—such as the brain and inner retina—exhibit strong autoregulation, maintaining microcirculatory perfusion at perfusion pressures above 50 to 60 mmHg.^6^^,^^7^ In sepsis, microcirculatory hypoperfusion, including within the cerebral circulation, occurs before macrocirculatory abnormalities, such as blood pressure, are detected^8^ and is linked to complications and mortality rates.^9^^,^^10^ This disconnect between macrocirculatory and microcirculatory perfusion is characteristic of sepsis and means that treatment which maintains systemic blood pressure may not preserve microcirculatory perfusion.^11^

Major surgery also challenges systemic perfusion with surgical stressors such as blood loss and inflammation, but unlike in sepsis, microcirculatory perfusion is relatively preserved.^12^

The retinal circulation supplies the inner retina,^13^ including the retinal ganglion cell layer (GCL), with retinal vascular abnormalities demonstrated in cerebrovascular and cardiac disease.^14^, ^15^, ^16^, ^17^ Animal and human studies of sepsis and shock report significant retinal vasculopathy and prolonged retinal arterial filling time on contrast angiography^18^, ^19^, ^20^ and reduced vascular length density of large retinal vessels on fundus photographs.^21^ OCT angiography (OCTA) is a superior and noninvasive technique to study retinal perfusion^22^^,^^23^ that is ubiquitous in ophthalmology but is less commonly applied in the intensive care unit (ICU), where it was feasible, reproducible, and reliable.^24^^,^^25^

We aimed to investigate alterations in retinal microvascular perfusion in patients in the ICU with sepsis in comparison to a control group of patients undergoing routine postoperative care in the ICU after major upper gastrointestinal surgery (ICU controls) and in a group of healthy controls. We hypothesized that, compared with healthy controls, retinal perfusion would be relatively preserved in patients undergoing uncomplicated postoperative care and be disrupted in patients with sepsis, leading to consequent inner retinal damage to the GCL as measured by OCT. Exploratory outcomes included the relationship between retinal perfusion and clinically relevant systemic measures, including the Sequential Organ Failure Assessment (SOFA) score.^26^

## Methods

### Study Design and Setting

A prospective observational cohort study evaluating alterations in retinal microvascular perfusion in patients in ICU with sepsis (n = 24) compared with an ICU control group of postoperative patients without sepsis who had undergone major surgery (n = 45) and a group of healthy controls (n = 15) was conducted between March 2018 and December 2022 in the Ophthalmology Department and Critical Care Unit at the Queen Elizabeth Hospital, part of University Hospitals Birmingham NHS Foundation Trust, Birmingham, United Kingdom. The study was approved by the East Midlands-Leicester South Research Ethics Committee (Reference, 14/EM/1163) and the Yorkshire and the Humber-Bradford Leeds Research Ethics Committee (Reference, 19/YH/0113). The study was conducted in accordance with the Declaration of Helsinki. Each subject signed an informed consent before participation in the ICU control and healthy control groups, including consent for publication. Nominated consultee declaration was sought in the sepsis group, with written informed consent signed by the patient if they recovered.

Adult patients who were scheduled for esophagectomy or gastrectomy surgery with planned postoperative ICU care were included in the study. As the primary surgery was elective, the research team was able to recruit eligible participants when they could consent. A healthy control group, with no prior history of eye or brain disease, cancer, or current infection, was recruited from among staff members. Sepsis was defined according to Sepsis-3 criteria, and patients were recruited if they had a SOFA score of ≥2 above baseline consequent to infection,^1^ and it was confirmed by the patient's treating physician.

Exclusion criteria included pre-existing retinal pathology, including a history of intraocular inflammation, diabetic macular edema, diabetic retinopathy, optic nerve pathology (including glaucoma), or known neurological conditions, assessed by history from patients, relatives, and the electronic medical record. Written informed consent was obtained from each ICU control and each healthy control subject before imaging. Patients with sepsis were unable to consent and were therefore recruited by a nominated consultee declaration obtained from the patient's next-of-kin or physician, and consent was sought from patients who recovered.

Sample size calculations were performed in G∗Power (Heinrich Heine University Düsseldorf).^27^ Based on initial evaluations showing grossly abnormal retinal perfusion in patients with sepsis, we assumed a large effect size compared with ICU controls (d = 0.8). A power calculation estimated that 19 patients admitted with sepsis and 39 ICU control patients without sepsis would give a power of 80% to detect a difference.

### OCT and OCTA Protocol

Where possible, patients in the ICU were imaged in the right eye first, then the left eye.

**Figure 1:**
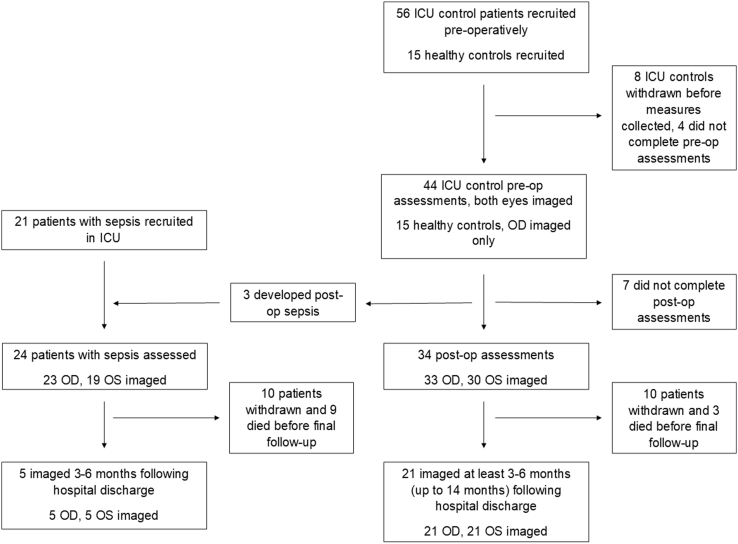
Study flowchart. ICU = intensive care unit; OD = right eye; OS = left eye; post-op = postoperative; pre-op = preoperative.

Healthy controls had OCT and OCTA imaging performed once in the right eye only. Intensive care unit control patients had OCT and OCTA imaging performed preoperatively, 24 to 48 hours postoperatively in the ICU, and 3 to 6 months postoperatively. Patients with sepsis received protocolized care according to the Surviving Sepsis Campaign,^28^ in addition to OCT and OCTA imaging, which was repeated 3 to 6 months after hospital discharge (Fig 1). If the pupil size limited scan quality, tropicamide 0.5% eye drop solution (Minims, Bausch & Lomb) was instilled. Likewise, surface lubricant hyaluronic acid (Hyloforte, Scope Ophthalmics Ltd) was applied where necessary as previously described.^24^

### Acquisition Devices

OCT and OCTA retinal images were acquired using the SPECTRALIS Heidelberg HRA + OCT (Heidelberg Engineering) mounted on the flex arm for assessments of patients on ICU and in supine healthy controls and on the table-top for preoperative and postoperative assessments. OCT angiography of the macula included 512 B-scans over an area of 3 × 3 mm, set at 4 automatic real-time averaging. The stability of taking these measures between different environments has been previously assessed.^29^ The use of TruTrack Active Eye Tracking ensured the reacquisition of images at the same retinal location in real time, ensuring that the follow-up images taken for each patient were at the same retinal location as previously imaged.

### OCT Analysis

Manufacturer's automated retinal layer segmentation was used to measure GCL and total retinal thickness in each ETDRS grid area.

### OCTA Analysis

Raw OCTA images were imported into the manufacturer's research software (SP-X1902, version 6.12.4.710, Heidelberg Engineering GmbH), and retinal layers were automatically segmented. One author (E.C.) manually verified retinal layer segmentation accuracy. Average perfusion within a macula 3 × 3 grid was automatically assessed and exported and summed to generate a final value for the macula superficial vascular plexus (SVP) and intermediate capillary plexus (ICP). Linked macula perfusion and GCL thickness were exported in ETDRS grid sections. The automatic segmentation matches that defined by Campbell et al*,*^30^ with the SVP spanning the GCL to midway of the inner plexiform layer, and the ICP spanning midway of the inner plexiform layer to midway of the inner nuclear layer. Metrics exported were sum (scaled amount of OCTA signal within a vasculature slab, representing the amount of perfusion in an area at that time; arbitrary units) and perfusion density (binary classification determined by whether the A-scan in the vasculature slab includes a perfused vessel; percentage value), as previously described.^25^^,^^29^^,^^31^

The foveal avascular zone (FAZ) area (mm^2^) was assessed using ImageJ Fiji (National Institutes of Health) exported as tag image file format OCTA en face images, with the scale set to 420 pixels/mm. The “polygon” setting was used to manually draw round the boundary of the FAZ to calculate area as previously described.^25^^,^^29^

### Visual Field Assessments

Visual field assessments were performed on a Humphrey visual field analyzer (Zeiss AG) using the Swedish Interactive Thresholding Algorithm 10-2 protocol preoperatively and at final follow-up in ICU control patients and at final follow-up in patients with sepsis.

### Systemic Assessments

Surrogate markers for microvascular dysfunction and proinflammatory states in critical illness were collected during inpatient OCTA assessment and for laboratory tests on the same day. The SOFA score was recorded at the time of OCTA imaging, including pO_2_/FiO_2_ (kPa), platelets (x10^3^/μL), bilirubin (μmol/L), mean arterial pressure (MAP; mmHg), Glasgow Coma Scale score (3–15), creatinine (μmol/L), and urine output (mL/d). Additional measures collected included C-reactive protein (mg/L), temperature (°C), respiratory rate (brpm), and heart rate (bpm). Vasopressor use and dose at the time of ICU imaging were also recorded.

### Statistics

Analyses were performed in IBM SPSS Statistics (IBM Corp, Released 2022. IBM SPSS Statistics for Windows, version 29. IBM Corp).

Differences between time points and between groups for retinal perfusion and thickness measurements, including data from both right and left eyes and different retinal areas for GCL analyses, as repeated measures, were modeled using generalized estimated equations with a linear model and an autoregressive correlation matrix. Demographic differences between groups were calculated using independent *t* test for numeric variables and chi-square with Yates correction for categorical variables.

Correlations between perfusion and systemic clinical and laboratory measures were assessed using Pearson *r* correlation.

## Results

Fifty-six patients who were due to have planned postoperative ICU care after esophagectomy or gastrectomy were recruited (42 male; 75%); 8 withdrew before any measures were collected as a result of changing surgical plans. All were upper gastrointestinal cancer patients, with 48 patients undergoing esophagectomy and 2 patients undergoing gastrectomy. Fifteen healthy controls were recruited. Twenty-one patients with sepsis were recruited (13 male, 63%). Three patients recruited before upper gastrointestinal surgery developed sepsis postoperatively and were imaged and analyzed as patients with sepsis, giving a total of 24 patients with sepsis. The number of patients imaged at each timepoint is reported in Figure 1. Because the coronavirus pandemic delayed final follow-up for some patients, time to follow-up varied between 3 and 14 months. Patient demographics and baseline characteristics are detailed in Table 1.

**Table 1:** Demographics and Clinical Characteristics of the Participants Recruited, Organized by Study Group

Variable	Healthy Control (n = 15)	ICU Control (n = 45)	Patients with Sepsis (n = 24)	*P* Value
Male, N (%)	11 (73)	36 (80.0)	14 (58.3)	0.10
Female, N (%)	4 (27)	9 (20.0)	10 (41.7)	0.10
Comorbidities, N (%)				
Diabetes mellitus	0	1 (1.8)	1 (4.2)	0.77
Ischemic heart disease	0	6 (11.3)	1 (4.2)	0.23
Cerebrovascular disease (strokes)	0	0 (0)	0 (0)	0.76
Malignancy	0	45 (100)	7 (29.2)	<0.001∗
Vasopressor use (%)	0	0	20 (83.3)	<0.001†
Age, mean (SD)	27.8 (10.86)	65.1 (9.3)	59.7 (11.3)	0.03‡
PO2/FiO2, kPa (SD)		36.8 (13.4)	32.1 (19.9)	0.163
Platelets, x10^3^/μL (SD)		246.9 (100.8)	182.3 (139.3)	0.002
Bilirubin, μmol/L (SD)		13.4 (11.2)	35.5 (60.4)	0.001
MAP, mmHg (SD)		87.1 (12.8)	74.5 (5.6)	0.000
Creatinine, μmol/L (SD)		70.4 (20.1)	102.8 (60.5)	0.000
CRP, mg/L (SD)		136.2 (91.2)	238.2 (134.8)	<0.001
Lactate, mmol/L (SD)		1.4 (0.5)	1.4 (0.8)	0.533
SOFA score (SD)		0.8 (1.2)	9.4 (3.9)	<0.001
Temperature, °C (SD)		36.5 (0.5)	36.7 (0.8)	0.271
Respiration rate, brpm (SD)		17.8 (3.0)	20.5 (3.6)	0.002
Heart rate, bpm (SD)		82.7 (14.0)	83.4 (16.3)	0.859
Hemoglobin, g/l (SD)		110.8 (17.8)	84.6 (18.1)	<0.001

brpm = breaths per minute; bpm = beats per minute; CRP = C-reactive protein; ICU = intensive care unit; MAP = mean arterial pressure; PO2/FiO2 = ratio of partial pressure of oxygen to fraction of inspired oxygen; SD = standard deviation; SOFA = Sequential Organ Failure Assessment.

Clinical measures were documented at the postoperative or septic timepoint. *P* values are given for the comparison between ICU control patients and patients with sepsis (chi-square test for categorical variables and independent *t* test for the remaining demographics).

∗ For the pairwise comparisons: ICU control vs. patients with sepsis, *P* < 0.001; patients with sepsis vs. healthy control, *P* = 0.02.

† For the pairwise comparisons: ICU control vs. patients with sepsis, *P* < 0.001; patients with sepsis vs. healthy control, *P* < 0.001.

‡ P value for ICU control vs. patients with sepsis; for ICU control vs. healthy control, *P* < 0.001.

### Effects of Sepsis on Retinal Perfusion

**Figure 2:**
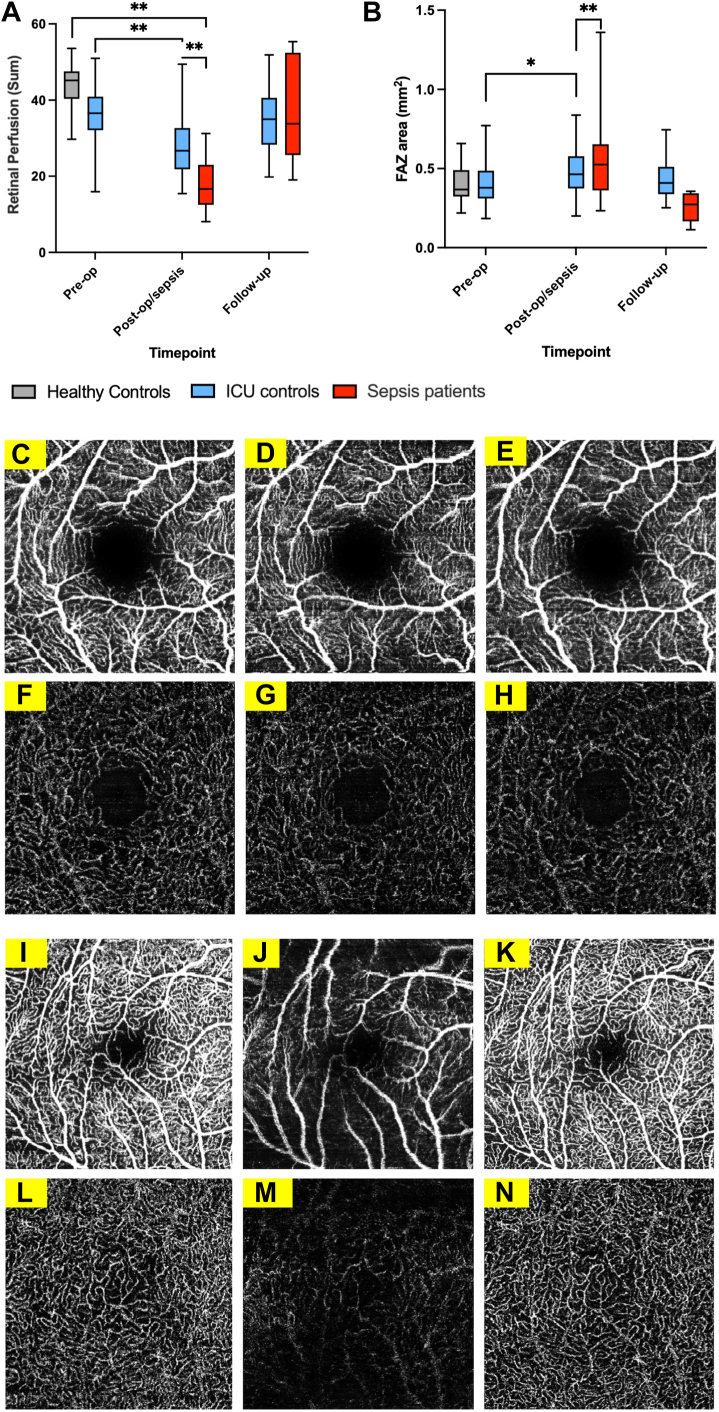
**A–B,** Box and whisker plots of macula SVP retinal perfusion at each timepoint for healthy controls, ICU controls, and patients with sepsis assessed by sum (**A**) and FAZ area (**B**). Healthy controls are shown in gray, ICU control patients are shown in blue, and patients with sepsis are shown in red. **C–H,** Representative OCTA images of the right eye SVP (**C–E)** and ICP (**F–H**) from an ICU control patient preoperatively (**C** and **F**), postoperatively (**D** and **G**), and at final follow-up (**E** and **H**). **I–N,** Representative OCTA images of the right eye SVP **(I–K)** and ICP **(L–N)** from a patient who developed sepsis after upper gastrointestinal cancer surgery, shown preoperatively (**I** and **L**), postoperatively with sepsis (**J** and **M**), and at final follow-up (**K** and **N**). ∗ = *P* < 0.05, ∗∗ = *P* < 0.001. Scale bar = 200 μm. At pre-op: ICU controls n = 44, healthy controls n = 15; at post-op/sepsis: ICU controls n = 34, sepsis n = 24; at follow-up: ICU controls n = 28, sepsis n = 7. FAZ = foveal avascular zone; follow-up = 3 to 6 month follow-up; ICP = intermediate capillary plexus; ICU = intensive care unit; OCTA = OCT angiography; pre-op = preoperative; post-op/sepsis = postoperative for intensive care unit control patients and inpatient care in intensive care unit for patients with sepsis; SVP = superficial vascular plexus.

Healthy control retinal perfusion measured by SVP sum was 35.7% higher (43.84 vs. 28.21; *P* < 0.001) than in ICU control patients, with similar results for all retinal perfusion measures in the SVP and ICP (Table S1, available at www.ophthalmologyscience.org), recovering by the final follow-up timepoint (Fig 2A and B, Table S1).

OCT angiography demonstrated lower retinal perfusion in patients with sepsis than in ICU control patients (Fig 2, Table S1, Figs S1 and S2, available at www.ophthalmologyscience.org). Superficial vascular plexus sum retinal perfusion was 37.4% lower in sepsis compared with ICU control patients (17.65 vs. 28.21, respectively, *P* < 0.001) and 59.7% lower than in healthy controls (43.84, *P* < 0.001). Retinal perfusion values recovered by the time of final follow-up in surviving patients. Representative images of retinal perfusion are shown in Figure 2.

### Association of Retinal Perfusion with Systemic Assessments and Vasopressor Use

**Figure 3:**
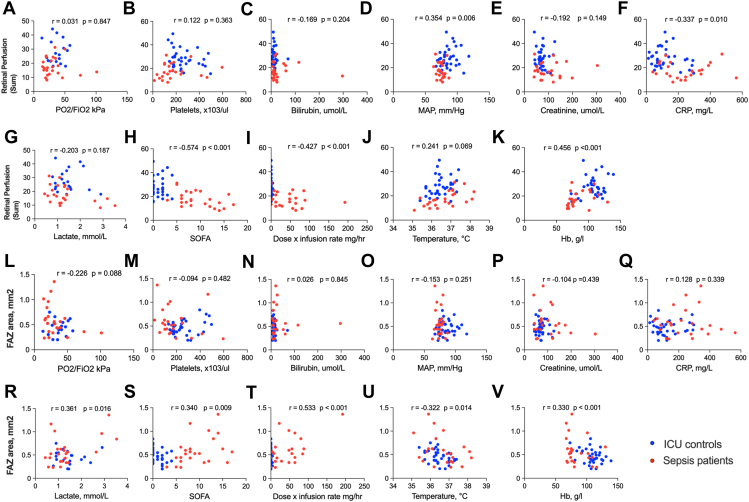
Scatter plots showing associations between macula SVP retinal perfusion and systemic clinical and laboratory measures, including vasopressor dose. Intensive care unit control patients are shown in blue; patients with sepsis are shown in red. **(A–K)** Sum retinal perfusion. **(L–V)** FAZ area. All Pearson *r* correlation values and corresponding *P* values are displayed for each plot. CRP = C-reactive protein; FAZ = foveal avascular zone; Hb = hemoglobin; ICU = intensive care unit; MAP = mean arterial pressure; PO2/FiO2 = ratio of partial pressure of oxygen to fraction of inspired oxygen; SOFA = Sequential Organ Failure Assessment; SVP = superficial vascular plexus.

Compared with ICU control patients, patients with sepsis had a higher SOFA score, with higher C-reactive protein and creatinine levels and lower platelets, hemoglobin, and MAP levels (Table 1). Superficial vascular plexus sum retinal perfusion was weakly correlated with MAP (*r* = 0.354, *P* = 0.006; Fig 3D) and C-reactive protein (*r* = −0.337, *P* = 0.010; Fig 3F) and moderately correlated with SOFA score (*r* = −0.574, *P* < 0.001; Fig 3H), with similar results in the ICP (Fig S3, available at www.ophthalmologyscience.org). The association between retinal perfusion and SOFA score was driven by patients with sepsis (ICU control *r* = −0.072, *P* = 0.687; sepsis *r* = −0.456, *P* = 0.025). Measured hemoglobin levels were strongly positively correlated with SVP sum retinal perfusion (*r* = 0.456, *P* < 0.001; Fig 3K) when analyzed across all patients, but not in the subgroup of patients with sepsis (SVP sum; *r* = 0.234, *P* = 0.134) and only weakly in ICU control patients (SVP sum *r* = 0.222, *P* = 0.08), suggesting that the correlation was driven by the difference between patients with sepsis and ICU controls. Age did not associate with retinal perfusion in the ICU (SVP sum *r* = 0.048, *P* = 0.627).

Retinal perfusion was negatively associated with vasopressor use and dose (Fig 3I, T; Fig S3I, T), with SVP sum decreasing (*r* = −0.427) and FAZ area increasing (*r* = 0.488) as vasopressor dose increased, although these relationships became nonsignificant when the single patient on 192 mg/hour of noradrenaline was excluded (sum *r* = −0.284, *P* = 0.190; FAZ *r* = 0.320, *P* = 0.137).

### Effect of Retinal Perfusion on Retinal GCL Thickness

**Figure 4:**
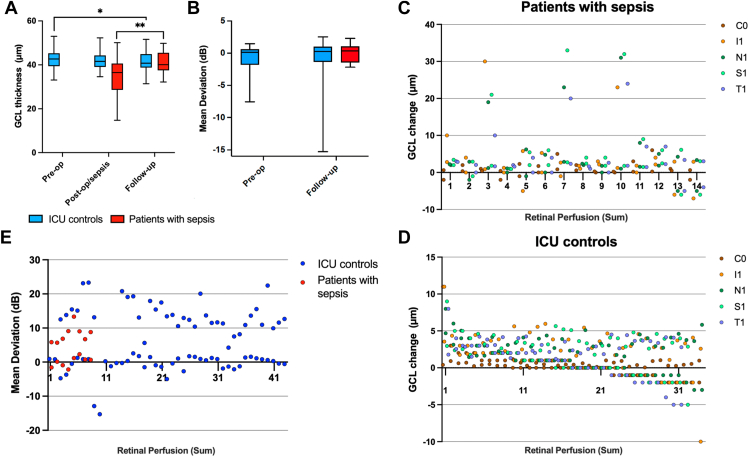
Changes in GCL thickness and Humphrey visual field MD. **(A)** Box and whisker plot of ETDRS grid area GCL thickness (μm) at each timepoint. **(B)** Box and whisker plot of MD (dB) at preoperative and follow-up timepoints only. Intensive care unit control patients are shown in blue; patients with sepsis are shown in red. **(C–D)** Scatter plots of the association between SVP sum retinal perfusion in ICU assessments and subsequent change in GCL thickness at follow-up in each central ETDRS grid area (central, C0; inferior, I1; nasal, N1; superior, S1; temporal, T1) in patients with sepsis **(C)** and ICU control patients **(D)**. At pre-op: ICU controls n = 44; at post-op/sepsis: ICU controls n = 34, sepsis n = 24; at follow-up: ICU controls n = 28, sepsis n = 7. **(E)** Scatter plot of the association between SVP sum retinal perfusion in ICU and MD at follow-up in both patients with sepsis and ICU control patients. At pre-op: n = 23; at follow-up: ICU control n = 21, sepsis n = 4. dB = decibels; GCL = ganglion cell layer; ICU = intensive care unit; MD = mean deviation; post-op/sepsis = postoperative for intensive care unit control patients and inpatient care in intensive care unit for patients with sepsis; pre-op = preoperative; SVP = superficial vascular plexus.

Compared with measurements taken in the ICU, GCL thickness across all patients increased by 2.94 ± 0.988 μm (*P* = 0.003; Fig. 4A) at final follow-up, driven by patients with sepsis, whose GCL increased by 6.71 ± 1.44 μm (*P* < 0.001) by the follow-up timepoint, compared with ICU control patients whose follow-up GCL thickness decreased by 0.128 ± 0.34 μm (*P* = 0.70). Compared with the preoperative timepoint, GCL thickness in ICU control patients at follow-up decreased by 0.823 ± 0.415 μm (*P* = 0.047).

Ganglion cell layer thickness changes at follow-up were strongly associated with retinal perfusion in ICU, with every 1-unit decrease in SVP sum predicting a 1.88 ± 0.634 μm increase in GCL thickness at follow-up (*P* = 0.003), with a greater effect of 3.50 ± 1.01 μm (*P* < 0.001; Fig. 4C) in patients with sepsis compared with 0.329 ± 0.164 μm (*P* = 0.045; Fig. 4D) in ICU control patients (0.199 ± 0.093 μm, *P* = 0.033, compared with the preoperative timepoint).

In contrast, total retinal thickness did not change significantly in ICU control patients between any timepoint (*P* = 0.122), but it decreased in patients with sepsis at the follow-up timepoint compared with measurements taken in ICU by 16.2 ± 5.76 μm, and this was not related to retinal perfusion (*P* = 0.208).

### Effect of Reduced Retinal Perfusion on Visual Function

Mean deviation (MD) on Humphrey visual field did not change between preoperative and follow-up timepoints in ICU control patients (*P* = 0.523; Fig. 4B) and was not different at the follow-up timepoint between patients with sepsis and ICU controls (*P* = 0.149). However, reduced retinal perfusion in the ICU was associated with MD at follow-up, with every 1-unit decrease in SVP sum predicting a 0.078 ± 0.034 decibel lower MD (*P* = 0.023; Fig. 4F), with similar subgroup effects of 0.120 ± 0.036 decibels in ICU control patients (*P* < 0.001) and 0.203 ± 0.076 decibels in patients with sepsis (*P* = 0.008).

## Discussion

This is the first report, to our knowledge, to show changes in retinal perfusion with major surgery and sepsis. Sepsis significantly reduced retinal perfusion, and these reductions in retinal perfusion measured in the ICU in both patients with sepsis and patients undergoing major surgery were associated with structural retinal changes and reduced visual function at follow-up.

Consistent with microcirculatory dysfunction being a known complication of sepsis,^8^, ^9^, ^10^^,^^23^^,^^32^ there was a weak relationship between retinal perfusion and MAP and a stronger association with SOFA score, indicating an association with organ dysfunction. The limited associations between retinal perfusion and systemic measures are consistent with the well-described dissociation between macrocirculatory parameters and microcirculatory health in sepsis.^33^, ^34^, ^35^

Patients with sepsis may rarely experience severe visual loss from ischemic optic neuropathy^36^ and retinal infarction, which might be expected from severe retinal ischemia and is not described. Studies of coronavirus disease 2019 do describe both retinal thickening and thinning,^37^^,^^38^ associated with the degree of hypoxia,^39^ and these findings are also reported in rodent models of intermittent hypoxia, although the determinants of whether the retina thins or thickens are unclear.^40^^,^^41^ OCT angiography assesses the inner retinal circulation (the outer retina being supplied by the choroid), and so changes in the GCL (inner retina) rather than the total retina are expected. It may be, therefore, that the retina suffers previously unrecognized damage as a result of sepsis, caused by hypoperfusion. Damage elsewhere in the CNS is well described in patients with sepsis, with neurocognitive frailty and postintensive care syndrome with neurocognitive decline after critical illness being a known complication,^42^^,^^43^ causing up to half of sepsis survivors long-term cognitive impairment termed “post-sepsis syndrome,”^44^^,^^45^ which may relate to the reported microvascular dysfunction.^8^, ^9^, ^10^^,^^23^^,^^32^

In ICU control patients, there was relatively preserved retinal perfusion and no relationship between retinal perfusion and SOFA score. Normal blood loss during an esophagectomy is between 225 and 800 ml,^46^ which would be consistent with the small reduction in retinal perfusion. The relative preservation of retinal perfusion seen in our study contrasts with more significant reductions in conjunctival and sublingual microvascular perfusion reported 6 to 8 hours after cardiac surgery,^47^ which may relate to different patient comorbidities, surgical stress, and experimental design (as we imaged after 24 hours), as well as stronger autoregulation present in the retina than peripheral circulations.^12^ However, the association between retinal perfusion and both GCL thickness and Humphrey visual field MD suggests that hypoperfusion during routine ICU care causes some damage to the retina. As follow-up was scheduled for 3 to 6 months postoperatively, the long-term effects of these changes have not been defined.

Other OCTA studies in coronavirus disease 2019 have reported long-term retinal microcirculatory compromise,^48^ but in contrast, our patients' retinal perfusion recovered at follow-up after both sepsis and major surgery. Although the mechanisms by which coronavirus disease 2019 causes microcirculatory dysfunction are unclear, the long-term compromise may relate to endothelial dysfunction caused by the infection. Many drivers of microcirculatory dysfunction in sepsis are part of the normal immune response to infection, and reversibility in response to acetylcholine infusion is described,^35^^,^^49^ which would be consistent with our results, as would an element of microcirculatory hypoperfusion caused by systemic microcirculatory compromise.

Limitations of this study include that it was observational and assessed a single timepoint during inpatient management, so it was not possible to monitor changes in retinal perfusion over time during ICU care. The healthy control group had the youngest age, fewest comorbidities, and highest retinal perfusion of any group, which could bias the assessment of normal retinal perfusion as higher than the ICU control patients at baseline. The cohort of patients with sepsis had fewer comorbidities and a lower mean age than the ICU control patients who were undergoing upper gastrointestinal cancer surgery, reflecting the difficulty in finding comparable control groups in an ICU environment, but nonetheless had very significantly reduced retinal perfusion in comparison. The high mortality and morbidity of both the sepsis and ICU control cohorts in a tertiary referral center limited the number of patients available for follow-up assessment of GCL thickness and visual fields, although the consistent effect across both cohorts supports our results. The study lacked monitoring of cardiac output or internal carotid artery Doppler as an assessment of large vessel blood supply to the retina and cannot therefore define the cause of reduced retinal perfusion as microvascular or global reductions in perfusion, although cerebral microcirculatory alterations in sepsis have been well-described,^50^^,^^51^ and MAP was maintained above what would ordinarily be the lower limit of ocular autoregulation, suggesting a degree of microvascular dysfunction.

Similarly, we did not exclude changes in intracranial pressure or intraocular pressure as contributing to the observed changes in retinal perfusion. While intracranial pressure does not directly affect retinal perfusion, there is a limited effect on retinal venous flow (explaining 9% of variation at elevated intracranial pressure).^52^ Intraocular pressure may be raised by 4 mmHg in patients with sepsis,^20^ which may also reduce retinal perfusion when autoregulation fails; however, the very marked changes seen in retinal perfusion in sepsis are very unlikely to be explained by such subtle changes in intraocular pressure or intracranial pressure.

## Conclusions

We report a measurable reduction in retinal perfusion in patients in the ICU with sepsis using OCTA and consequent changes in retinal structure and reductions in visual function in both patients with sepsis and patients having major surgery. The association with SOFA score, and weaker association with other global perfusion measures, would be consistent with OCTA assessing sepsis-induced microcirculatory hypoperfusion.
