# Supplementary material for: Retinal Perfusion and Injury in Sepsis and after Major Surgery
Source: Ophthalmol Sci. 2025 Jul 22;6(1):100890. doi: 10.1016/j.xops.2025.100890 (PMC12481890; doi:10.1016/j.xops.2025.100890)
Supplement: Table S1 [file mmc1.pdf]

| Vessel layer | Perfusion measure | Group | Timepoint | Modelled value | SE | 95% CI | P-value |
|---|---|---|---|---|---|---|---|
| **Superficial vascular plexus** | **Sum** | Healthy controls | | 43.84 | 1.56 | 40.72 to 46.96 | <0.001 (vs post-op) |
| | | ICU Controls | Pre-op<br>Post-op<br>Follow-up | 35.32<br>28.21<br>34.42 | 1.258<br>1.042<br>1.429 | 30.056 to 40.59<br>26.17 to 30.25<br>31.615 to 37.22 | <br><0.001 (vs pre-op)<br>0.471 (vs pre-op) |
| | | Sepsis patients | ICU<br>Follow-up | 17.65<br>38.18 | 1.648<br>5.722 | 12.38 to 22.92<br>26.97 to 49.40 | <0.001 (vs control) |
| | **Perfusion density (%)** | Healthy Controls | | 30.17 | 1.12 | 27.93 to 32.41 | <0.001 (vs post-op) |
| | | ICU Controls | Pre-op<br>Post-op<br>Follow-up | 28.42<br>23.28<br>27.47 | 0.921<br>0.936<br>1.063 | 26.56 to 30.23<br>19.64 to 26.92<br>25.38 to 29.56 | <br><0.001 (vs pre-op)<br>0.319 (vs pre-op) |
| | | Sepsis patients | ICU<br>Follow-up | 140.9<br>266.7 | 11.06<br>27.79 | 105.6 to 176.3<br>212.3 to 321.2 | <0.001 (vs control) |
| | **FAZ area ($mm^2$)** | Healthy Controls | | 0.400 | 0.030 | 0.341 to 0.459 | 0.104 (vs post-op) |
| | | ICU Controls | Pre-op<br>Post-op<br>Follow up | 0.421<br>0.463<br>0.428 | 0.021<br>0.030<br>0.013 | 0.379 to 0.462<br>0.405 to 0.522<br>0.360 to 0.494 | <br>0.042 (vs pre-op)<br>0.613 (vs pre-op) |
| | | Sepsis patients | ICU<br>Follow-up | 0.582<br>0.458 | 0.077<br>0.062 | 0.431 to 0.734<br>0.336 to 0.579 | <0.001 (vs control) |
| **Intermediate capillary plexus** | **Sum** | Healthy Controls | | 23.29 | 0.72 | 24.73 to 21.85 | <0.001 (vs post-op) |
| | | ICU Controls | Pre-op<br>Post-op<br>Follow-up | 19.27<br>14.30<br>18.91 | 0.689<br>0.641<br>0.871 | 17.92 to 20.62<br>13.04 to 15.55<br>17.21 to 20.62 | <br><0.001 (vs pre-op)<br>0.624 (vs pre-op) |
| | | Sepsis patients | ICU<br>Follow-up | 9.196<br>19.07 | 0.926<br>2.373 | 7.381 to 11.01<br>14.42 to 23.72 | <0.001 (vs control) |
| | **Perfusion density (%)** | Healthy Controls | | 39.67 | 1.004 | 41.68 to 37.66 | <0.001 (vs post-op) |
| | | ICU Controls | Pre-op<br>Post-op<br>Follow-up | 35.73<br>28.41<br>34.62 | 0.997<br>1.119<br>1.023 | 33.78 to 37.68<br>26.22 to 30.6<br>32.61 to 36.62 | <br><0.001 (vs pre-op)<br>0.285 (vs pre-op) |
| | | Sepsis patients | ICU<br>Follow-up | 148.6<br>312.9 | 12.57<br>27.06 | 124.0 to 173.2<br>259.8 to 365.9 | <0.001 (vs control) |
| | **FAZ area ($mm^2$)** | ICU Controls | Pre-op<br>Post-op<br>Follow-up | 0.269<br>0.284<br>0.273 | 0.017<br>0.018<br>0.017 | 0.235 to 0.303<br>0.248 to 0.320<br>0.239 to 0.307 | <br>0.315 (vs pre-op)<br>0.340 (vs pre-op) |
| | | Sepsis patients | ICU<br>Follow-up | 0.330<br>0.249 | 0.036<br>0.035 | 0.259 to 0.400<br>0.181 to 0.318 | 0.410 (vs control) |

**Supplementary Table 1**. Superficial vascular plexus and intermediate capillary plexus retinal perfusion values at pre-operative (pre-op), immediately post-operative (post-op) and 3-6 month follow-up (follow-up) timepoints in the ICU control group (upper gastrointestinal patients who did not develop sepsis), and in patients with sepsis at ICU and 3-6 month follow-up (follow-up). P values are given for the comparison with pre-op for control patients and for the comparison with post-op controls for patients with sepsis. Modelled mean values, p values and 95% confidence intervals are from generalized estimating equations. Abbreviations: SE: standard error; CI: confidence interval; ICU: intensive care unit; FAZ: foveal avascular zone.
