# Supplementary material for: Retinal Perfusion and Injury in Sepsis and after Major Surgery
Source: Ophthalmol Sci. 2025 Jul 22;6(1):100890. doi: 10.1016/j.xops.2025.100890 (PMC12481890; doi:10.1016/j.xops.2025.100890)
Supplement: Figure S1 [file mmc2.pdf]

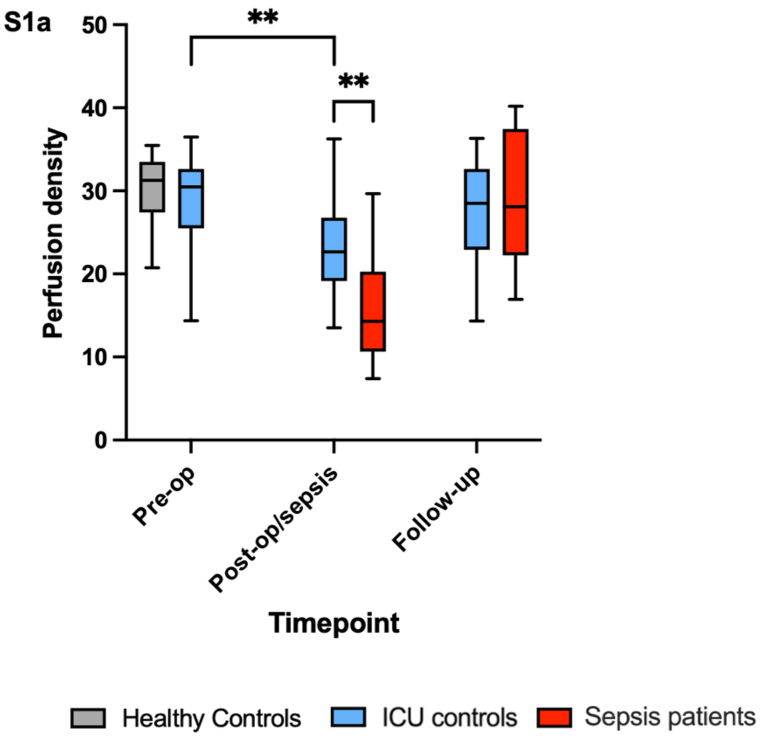

Supplementary Figure 1. Box and whisker plots to show SVP perfusion density (%) retinal perfusion at each timepoint for healthy controls (shown in grey), ICU controls (shown in blue) and sepsis patients (shown in red). **= $p<0.001$. At pre-op timepoint: healthy controls n=15, ICU controls n=44; at post-op / sepsis timepoint: ICU controls n=34, sepsis patients =24; at follow-up: ICU controls n=21, sepsis patients n=7. Abbreviations: ICU: intensive care unit; SVP: superficial vascular plexus; ICP: intermediate capillary plexus; pre-op: pre-operative; post-op: post-operative.
