# Supplementary material for: Retinal Perfusion and Injury in Sepsis and after Major Surgery
Source: Ophthalmol Sci. 2025 Jul 22;6(1):100890. doi: 10.1016/j.xops.2025.100890 (PMC12481890; doi:10.1016/j.xops.2025.100890)
Supplement: Figure S2 [file mmc3.pdf]

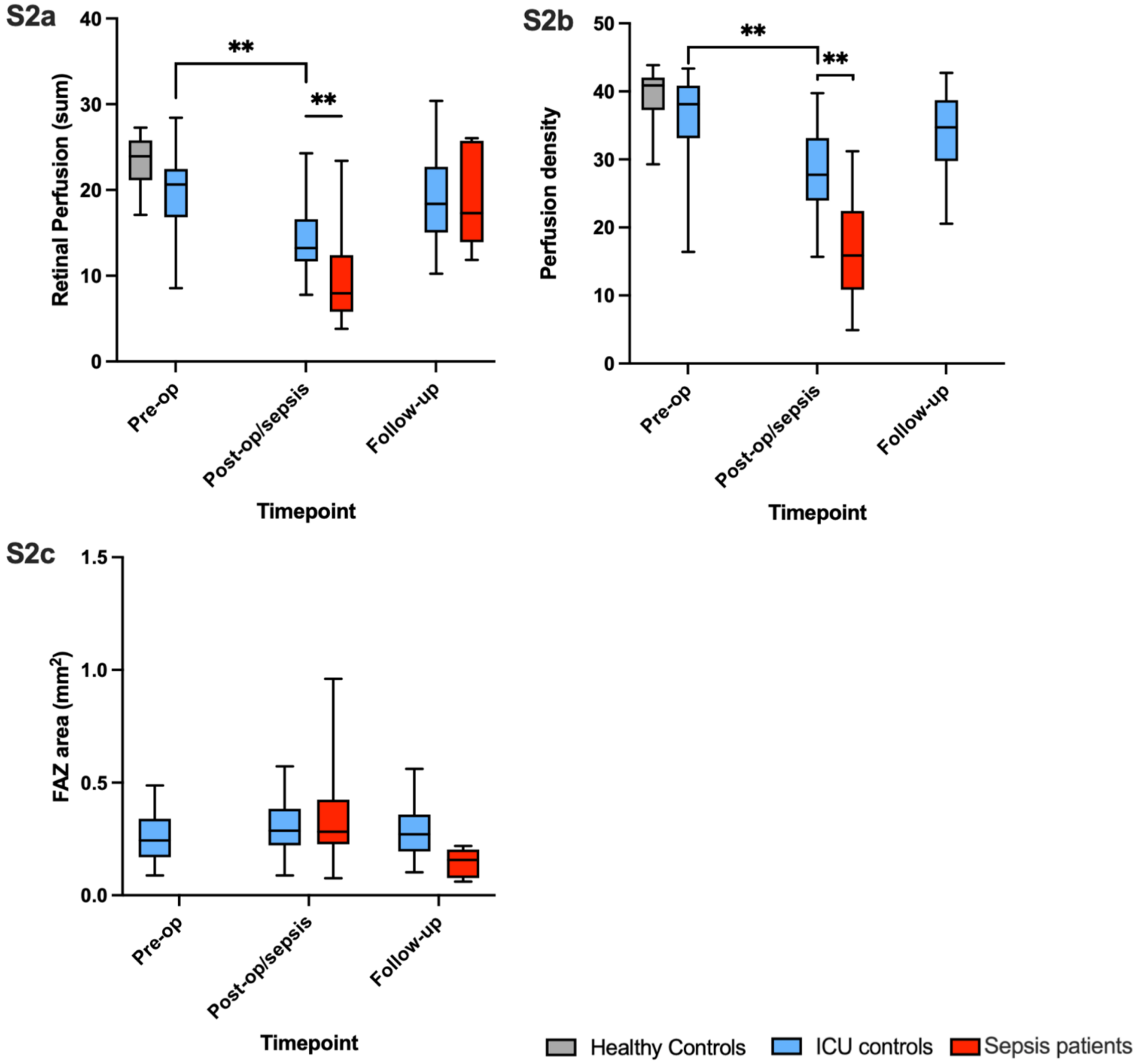

Supplementary Figure 2. Box and whisker plots of ICP retinal perfusion at each timepoint for healthy controls (shown in grey), ICU controls (shown in blue) and sepsis patients (shown in red). **S2a)** Sum. **S2b)** Perfusion density (%). **S2c)** FAZ area (mm^2). **=p<0.001. At pre-op timepoint: healthy controls n=15, ICU controls n=44; at post-op / sepsis timepoint: ICU controls n=34, sepsis patients =24; at follow up: ICU controls n=21, sepsis patients n=7. Abbreviations: ICP: intermediate capillary plexus; ICU: intensive care unit; FAZ: foveal avascular zone
