# Supplementary material for: Retinal Perfusion and Injury in Sepsis and after Major Surgery
Source: Ophthalmol Sci. 2025 Jul 22;6(1):100890. doi: 10.1016/j.xops.2025.100890 (PMC12481890; doi:10.1016/j.xops.2025.100890)
Supplement: Figure S3 [file mmc4.pdf]

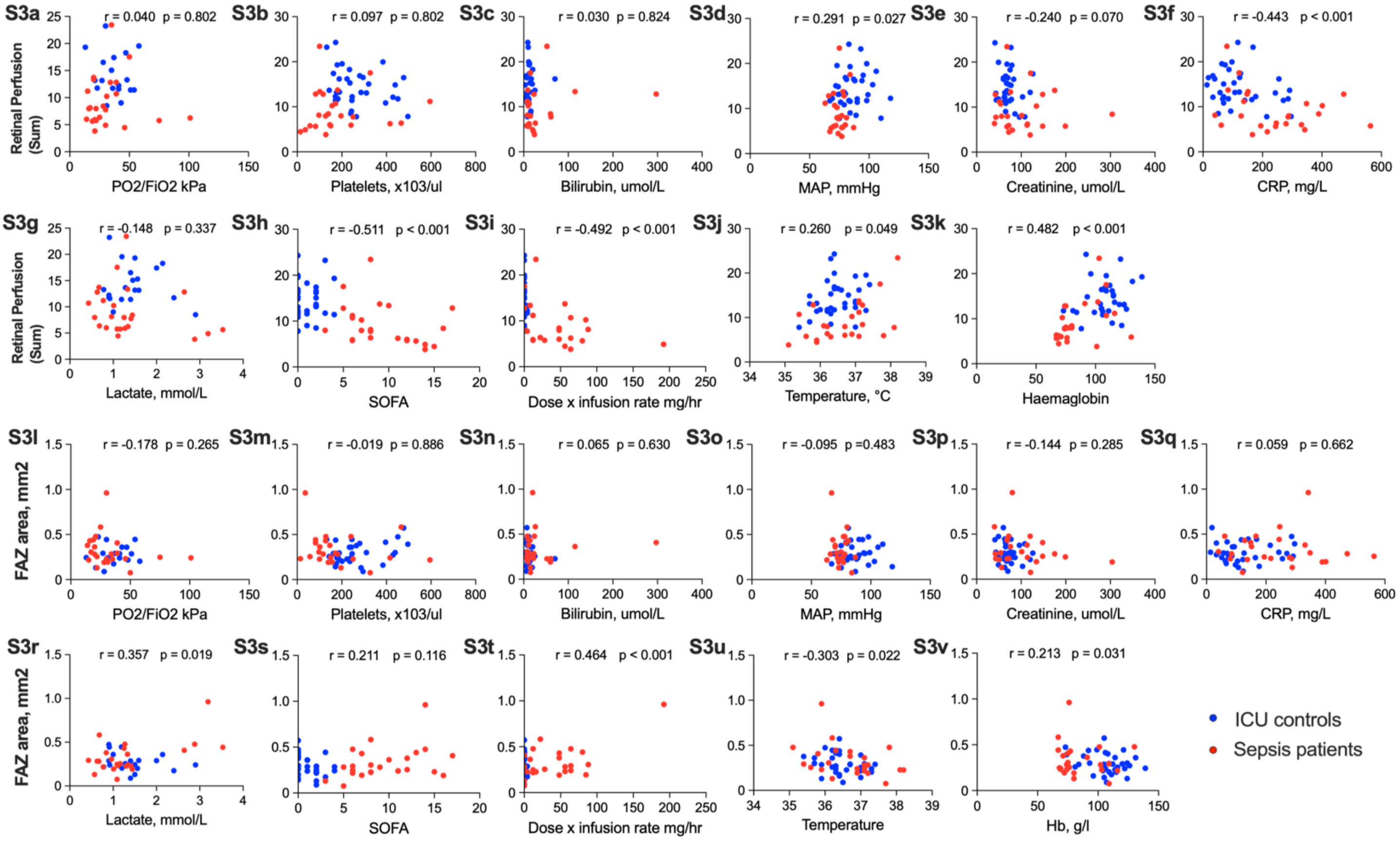

Supplementary Figure 3. Scatter plots of associations between ICP retinal perfusion and systemic clinical and laboratory measures, including vasopressor dose. **S3a-l)** ICP sum associations**. S3m-v)** FAZ area (mm^2) associations. Pearson's r correlation values and corresponding p-values are displayed for each plot. Abbreviations: MAP: mean arterial pressure; CRP: C-reactive protein; SOFA: Sequential [Sepsis-Related] Organ Failure Assessment; FAZ: foveal avascular zone; ICU: intensive care unit; ICP: intermediate capillary plexus.
